# Vessel-wall imaging and quantification of flow-mediated dilation using water-selective 3D SSFP-echo

**DOI:** 10.1186/1532-429X-15-100

**Published:** 2013-10-30

**Authors:** Michael C Langham, Cheng Li, Erin K Englund, Erica N Chirico, Emile R Mohler, Thomas F Floyd, Felix W Wehrli

**Affiliations:** 1 Department of Radiology, School of Medicine, University of Pennsylvania, Philadelphia, USA; 2 Department of Medicine, School of Medicine, University of Pennsylvania, Philadelphia, USA; 3 Departments of Anesthesiology, Medical Center, Stony Brook University, Stony Brook, USA; 4 Radiologic Science, Biochemistry and Biophysics, Medical Center, University of Pennsylvania, 3400 Spruce Street, Philadelphia, PA 19104, USA

## Abstract

**Background:**

To introduce a new, efficient method for vessel-wall imaging of carotid and peripheral arteries by means of a flow-sensitive 3D water-selective SSFP-echo pulse sequence.

**Methods:**

Periodic applications of RF pulses will generate two transverse steady states, immediately after and before an RF pulse; the latter being referred to as the SSFP-echo. The SSFP-echo signal for water protons in blood is spoiled as a result of moving spins losing phase coherence in the presence of a gradient pulse along the flow direction. Bloch equation simulations were performed over a wide range of velocities to evaluate the flow sensitivity of the SSFP-echo signal. Vessel walls of carotid and femoral and popliteal arteries were imaged at 3 T. In two patients with peripheral artery disease the femoral arteries were imaged bilaterally to demonstrate method’s potential to visualize atherosclerotic plaques. The method was also evaluated as a means to measure femoral artery flow-mediated dilation (FMD) in response to cuff-induced ischemia in four subjects.

**Results:**

The SSFP-echo pulse sequence, which does not have a dedicated blood signal suppression preparation, achieved low blood signal permitting discrimination of the carotid and peripheral arterial walls with in-plane spatial resolution ranging from 0.5 to 0.69 mm and slice thickness of 2 to 3 mm, i.e. comparable to conventional 2D vessel-wall imaging techniques. The results of the simulations were in good agreement with analytical solution and observations for both vascular territories examined. Scan time ranged from 2.5 to 5 s per slice yielding a contrast-to-noise ratio between the vessel wall and lumen from 3.5 to 17. Mean femoral FMD in the four subjects was 9%, in good qualitative agreement with literature values.

**Conclusions:**

Water-selective 3D SSFP-echo pulse sequence is a potential alternative to 2D vessel-wall imaging. The proposed method is fast, robust, applicable to a wide range of flow velocities, and straightforward to implement.

## Background

The most common site of cerebrovascular atherosclerotic disease is the outer wall of the carotid sinus proximal to the bifurcation. At arterial bifurcations or regions of high curvature the unidirectional flow gives way to complex hemodynamics including reversing flow patterns leading to low wall shear stress, a condition promoting atherogenesis [1,2]. Much of the work on the high-resolution vessel-wall, black-blood cardiovascular magnetic resonance (CMR) has focused on carotid artery disease and demonstrated to be a powerful method for assessment of plaque burden [3,4]. In comparison only a few studies exist targeting peripheral arteries at 3 T, e.g. common femoral and popliteal arteries [5,6]. In patients with peripheral artery disease (PAD) the image-based evaluation can be crucial, especially when revascularization is being considered. Unlike the contrast-enhanced MRA and x-ray angiography black-blood CMR does not expose the patient to ionizing radiation or gadolinium-based contrast agents. The latter is problematic with patients with PAD who often have impaired renal function [7,8]. However, the major challenge for peripheral arteries is the need for larger anatomic coverage, thus an efficient acquisition technique is needed. In addition to assessment of vessel-wall morphology, a rapid black-blood technique has the potential to quantify flow-mediated dilation (FMD), a surrogate marker of endothelial function [9,10], in peripheral arteries in response to a challenge, e.g. arterial occlusion.

The double inversion recovery (DIR) pulse sequence [11] with sequential 2D readout is the most widely used method to suppress blood signal in the lumen. The principle of the method is to detect the signal at or near the null-point of the longitudinal magnetization recovery of blood after back-to-back non-selective and slice-selective inversions. The slice-selective inversion restores the magnetization of the tissue protons while the blood in the imaging slice is refreshed with blood that has null signal. Alternate approaches to blood signal suppression include motion-sensitized driven equilibrium (MSDE) [12] and saturation bands above and below the imaging slice. In MSDE intravoxel dephasing of the blood signal is achieved with bipolar gradients in a T2-preparation module and a final 90° pulse restoring the tissue magnetization. An alternative to DIR or MSDE approach to blood signal suppression is the flow-sensitive pulse sequence of 3D turbo spin-echo with variable flip-angle refocusing RF pulses [13,14]. In this approach the readout direction is parallel to the flow direction and the non-180° refocusing pulses generate stimulated echoes of varying phase causing intravoxel dephasing from destructive interference of spin isochromats.

In this work we present an alternative approach to 2D vessel-wall imaging of carotid and peripheral arteries at 3 T using a rapid 3D water-selective SSFP-echo pulse sequence without a blood-signal suppression preparation module as in MSDE or DIR. In a SSFP pulse sequence the periodic application of RF pulses leads to formation of two transverse magnetization steady states [15,16], immediately after and before each RF pulse, referred to as SSFP-fid and SSFP-echo, respectively. For flowing spins the transverse steady state is disrupted because the advancing spins cause different phase accumulation between pulse cycles [17]. In brief, the flow sensitivity of SSFP-echo can be understood by considering that the detected signal is a superposition of the refocused FID caused by multiple preceding RF pulses. The multitude of coherence pathways from magnetization excited by prior RF pulses results in superposition of echoes with incoherent phases. Thus, the mechanism for blood signal suppression of the SSFP-echo pulse sequence is similar to the one operating in SPACE since non-180° refocusing pulses generate multiple pathways that interfere destructively at any given echo. Here we describe the basic structure of the SSFP-echo pulse sequence, provide details of its flow sensitivity and demonstrate the pulse sequence’s potential as a viable vessel-wall imaging technique for carotid and peripheral arteries and as a potential means for quantifying flow-mediated dilation.

## Methods

### Flow sensitivity and optimization of water-selective 3D SSFP-echo

**Figure 1:**
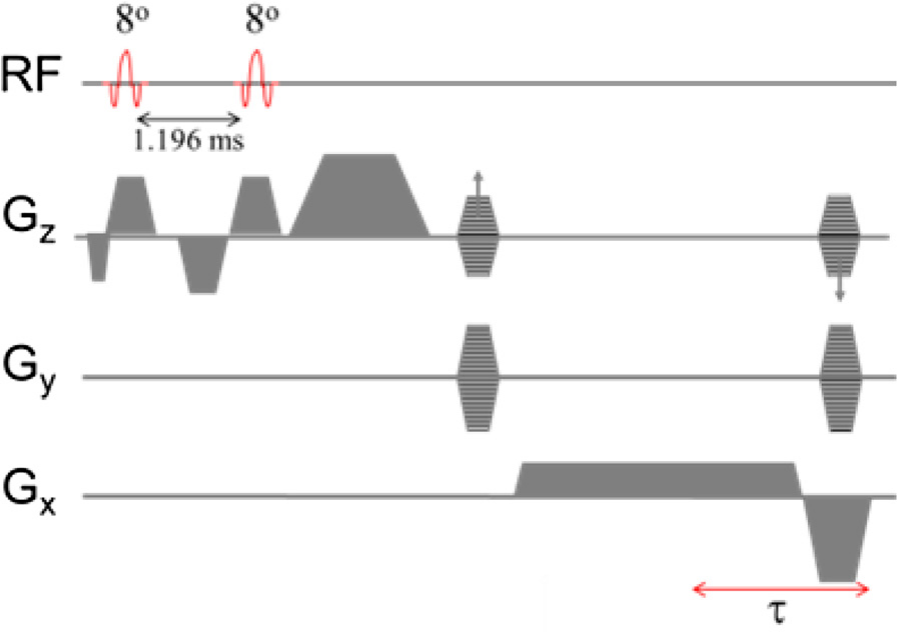
**Water-selective 3D SSFP-echo pulse sequence.** SSFP-echo can be thought of as a time-reversed GRE pulse sequence with TE = 2TR-τ. The effective flip angle of the binomial pulses is 16° and 0° for water and fat protons, respectively. See text for details.

**Figure 2:**
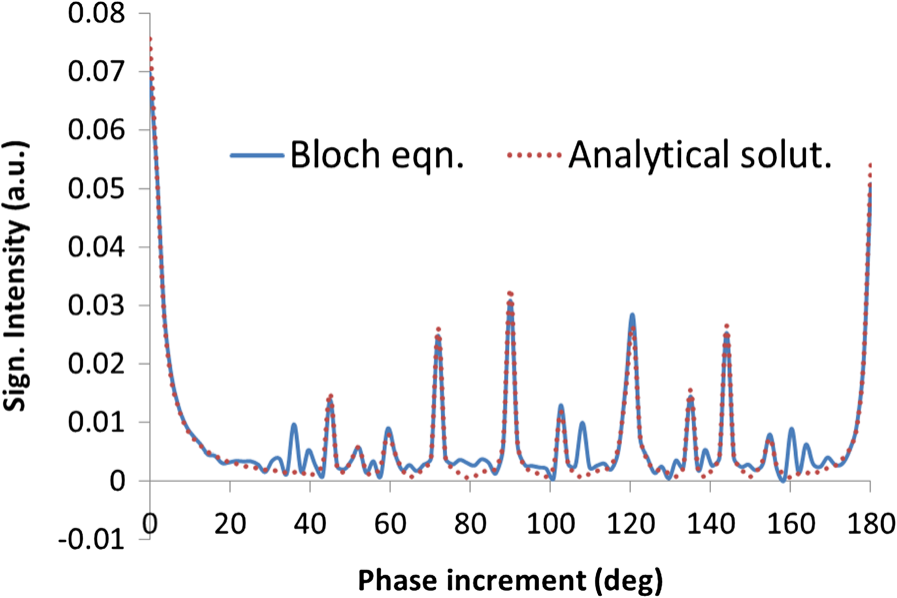
**Transverse SSFP-echo signal dependence on RF phase increment ϕ.** Comparison between Bloch equation simulation and analytical solution of RF-spoiled SSFP-echo is shown. T1/T2/TR = 1850/150/8.9 ms were used in the simulation.

Figure 1 shows a water-selective 3D SSFP-echo pulse sequence. Fat suppression is achieved with a 1–1 binomial pulse, in which the two successive pulses are separated by about 1.2 ms, which is approximately equal to the half-period of the fat-water chemical shift difference at 3 T. The 2nd RF pulse restores the magnetization of the fat CH_2_ protons along the z-axis. Bloch equation simulations based on the relaxation characteristics for muscle tissue (T1/T2 = 1450/25 ms) predict an optimal flip angle 8° + 8°. A crusher gradient parallel to the blood flow direction is applied immediately after the 2nd RF pulse to suppress the FID and impart phase to the spins in blood. The crusher gradient’s 0th moment is set to 24 mT⋅ms/m, which will impart 4π phase dispersion along the slice-direction with 2 mm resolution. The flow sensitivity of SSFP-echo can be understood in terms of RF spoiling [17,18] induced by bulk flow, which we will refer to as flow-induced RF spoiling (RF_fl_). Unlike static spins, the phases of the moving spins become incoherent from the displacement in the presence of a gradient pulses along the flow direction (here the FID crusher gradient, Figure 1). Dephasing of spins moving perpendicular to the major flow direction is imparted by the initial portion of the readout gradient as well as by the centric phase-encoding scheme that causes larger jumps in flow-induced phase between consecutive phase-encoding steps [19]. In a spoiled GRE pulse sequence the RF phase increases as *φ*_*k*_ - *φ*_*k*-1_ = *k*ϕ [20], where *φ*_*k*_ represents the RF phase of the k^th^ pulse sequence cycle and ϕ is the phase increment. The recommended ϕ is 117° to suppress steady-state transverse magnetization over a wide range of T1, T2 and flip angles [20]. In RF_fl_ spoiling the phase increment is generated by the displacement of the spins between pulse sequence cycles rather than from non-zero first moment that results in the presence of a gradient along the flow direction. Thus constant velocity can also lead to RF_fl_ spoiling or quadratic increase in RF phase. For example, in the first pulse sequence cycle the phase accumulation can be expressed as

(1) $$\phi_{1}=\gamma \int G\left(r_{o}+v_{o}t\right)\mathrm{dt},$$

where G is the amplitude of the gradient and r_o_ and v_o_ are the initial position and velocity of the spins, respectively. For the following cycle the phase is

(2) $$\phi_{2}=\phi_{1}+\gamma \int G\left(r_{o}+v_{o}\text{TR}+v_{o}t\right)\text{dt}.$$

as spins are displaced by *v*_*o*_*TR*. After the n^th^ cycle the spin will be displaced by *nv*_*o*_*TR* from the initial position. Thus, the phase after the n^th^ cycle is

(3) $$\begin{array}{l} \phi_{n}=\phi_{n-1}+\gamma \int G\left(r_{o}+nv_{o}\text{TR}+v_{o}t\right)\text{dt},\;\mathrm{or}\\ \Delta \phi =\phi_{n}-\phi_{n-1}=\phi_{1}+n\gamma Gv_{o}\text{TRδ}, \end{array}$$

where δ is the duration of the gradient [17]. In reality, the phase increment *ϕ* in RF_fl_ spoiling will vary from pulse cycle to cycle due to non-constant velocity and will be minimal during diastole. However, Bloch equation simulations(Figure 2) indicate that even a small phase increment *ϕ* can significantly attenuate the longitudinal magnetization and spoil the transverse magnetization and a large phase increment is not necessarily superior (c.f. local peaks occur for *ϕ* > 70°). The analytical solution of SSFP-echo signals with RF spoiling [21,22] is shown for reference (Figure 2). RF spoiling is less effective for phase increments of 45°, 72°, 90°, 120°, etc., which are integral fractions of 360°.

### Velocity waveforms and effective phase increments of RF_f_ spoiling

The carotid arteries feed low vascular-resistance beds, thus the velocity waveform is monophasic. The time-resolved velocity averaged across the lumen ranges from about 15 to 50 cm/s and the velocity averaged over the cardiac cycle is approximately 25 to 35 cm/s. In our implementation the 0^th^ moment of the FID crusher is 24 mT⋅ms/m and the corresponding phase increment from displacement *γm*_*o*_*v*_*o*_*TRδ* (*mod* 2π) is 4.14 rads if we take the average velocity (averaged over the cardiac cycle and lumen) as v_o_ = 30 cm/s. Thus, the resulting phase increment leads to substantial attenuation of longitudinal magnetization even from low velocities as shown in [23] and Figure 2, e.g. at 1 cm/s the corresponding phase increment is 0.138 rad or 7.9°.

The femoral and popliteal arteries feed high vascular-resistance bed and the velocity wave-form is triphasic with retrograde flow following after the systolic peak and the end-diastolic flow is characterized by low-amplitude antegrade flow. The average peak systolic velocity can also reach up to 50 cm/s but due to the retrograde flow and long diastolic period the average velocity over the cardiac cycle is typically less than 5 cm/s. Thus the average phase increment is approximately 39°.

### Imaging

Written informed consent was obtained prior to all human studies following the protocol approved by the Institutional Review Board of the University of Pennsylvania. In all studies a 3D water-selective SSFP-echo sequence was used to acquire axial images without cardiac gating on a Siemens 3 T TIM Trio scanner.

#### ***Carotid arteries***

The carotid arteries were imaged in six healthy subjects (ages 25 – 41 years, average 30.8 ± 8.3 yrs) with custom-built bilateral dual-element phased-array carotid coils [24]: flip angle = 8° + 8°, TE/TR = 3.9/8.7 ms, bandwidth = 219 Hz/voxel, FOV = 140 × 128 × 50 mm^3^, spatial resolution 0.61 × 0.61 × 2 mm^3^, number of signal averages = 3(acquired sequentially), total acquisition time 140 s.

#### ***Peripheral arteries***

The femoropopliteal arteries of ten healthy subjects (ages 25–65, average age 43.5 ± 13.6 yrs) were imaged using an 8 channel transmit/receive extremity coil (Invivo Inc., Pewaukee, WI) using the same flip angle and TE/TR but with bandwidth = 178 Hz/voxel, larger slab thickness FOV = 140 × 128 × 192 mm^3^, higher in-plane resolution = 0.5 × 0.5 × 2 mm^3^ and no signal averaging; resulting in a total acquisition time of less than 4 mins. The manufacturer’s spine coil combined with the body matrix coils (placed over the lower abdomen and thighs) were used to image the deep and superficial femoral arteries below the bifurcation, along with the following imaging parameters: flip angle = 8° + 8°, TE/TR = 4.35/9.42 ms, bandwidth = 195 Hz/voxel, FOV = 352 × 192 × 360 mm^3^, spatial resolution = 0.69 × 0.69 × 3 mm^3^, no signal averaging, total acquisition time = 5 mins 15 s. In two patients with unilateral PAD (ABI = 0.65 and 0.55; the ankle-brachial index (ABI) was obtained as the ratio between systolic pressures of the ankle, and brachial artery), deep femoral artery(DFA) and superficial femoral artery (SFA) were imaged bilaterally to visualize both the diseased and healthy arteries for the purpose of demonstrating the method’s potential to visualize atherosclerotic plaques.

#### ***Flow-mediated dilation (FMD) of femoral artery***

FMD (defined as the fractional change in vessel diameter) is traditionally measured on the brachial artery to assess endothelial function [10] but atherosclerosis in the upper extremity is rare [25]. FMD of femoral or popliteal artery may potentially be a better marker of endothelial dysfunction since there is a greater propensity to develop vascular pathology in the legs [26].

To demonstrate the feasibility of femoral artery FMD quantification vessel-wall images were acquired in four subjects (ages 27, 33, 41 and 54 yrs) at rest and at 90 s after the cuff deflation following 5 mins of cuff occlusion applied on the upper thigh. Arterial occlusion was achieved by inflating the cuff 75 mmHg above the subject’s systolic pressure but not exceeding 250 mmHg. As a part of a localizer scan 3D time-of-flight MRA was used to ensure that the imaging slab was locally perpendicular to the femoral artery. The 8-channel extremity coil was used with the following imaging parameters: flip angle = 8° + 8°, TE/TR = 3.99/9.97 ms, bandwidth = 223 Hz/voxel, FOV = 128 × 128 × 21 mm^3^, spatial resolution = 0.8 × 0.8 × 3 mm^3^(zero padded to 0.4 × 0.4 mm^2^), no signal averaging, total acquisition time <12 s (temporal resolution).

### SNR, CNR and FMD_A_ measurements

The SSFP-echo is an echo-shifted pulse sequence in which TE is greater than TR since the primary contribution to the signal is the partially refocused FID from the previous pulse cycle. Due to limited slab thickness signal averaging (N = 3) was used for the carotid wall imaging to enhance SNR. The repeats were acquired sequentially and the final image was reconstructed by taking the absolute value of the complex mean to further attenuate the blood signal via destructive interference.

SNR of vessel wall and lumen was computed as SI/σ where SI is the signal intensity of the ROI and σ is the standard deviation of noise, determined from a region in the air surrounding the neck or leg. CNR was defined as the SNR difference between the vessel wall and lumen and CNR efficiency was computed as CNR divided by the product of voxel volume and square root of the average acquisition time per slice.

For the flow-mediated dilation quantification the lumen area was estimated with an automatic segmentation algorithm where the threshold level is set by the mean signal in the lumen [27]. The fractional change in the vessel lumen area (FMD_A_) was computed instead of the diameter, as typically practiced in ultrasound.

## Results

**Figure 3:**
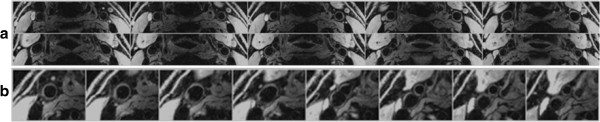
**Representative contiguous slices about the carotid bifurcation from two healthy subjects. a)** Seven bilateral images from a 26 year-old subject. **b)** Eight magnified views of the right carotid artery of a 41 year-old subject.

**Figure 4:**
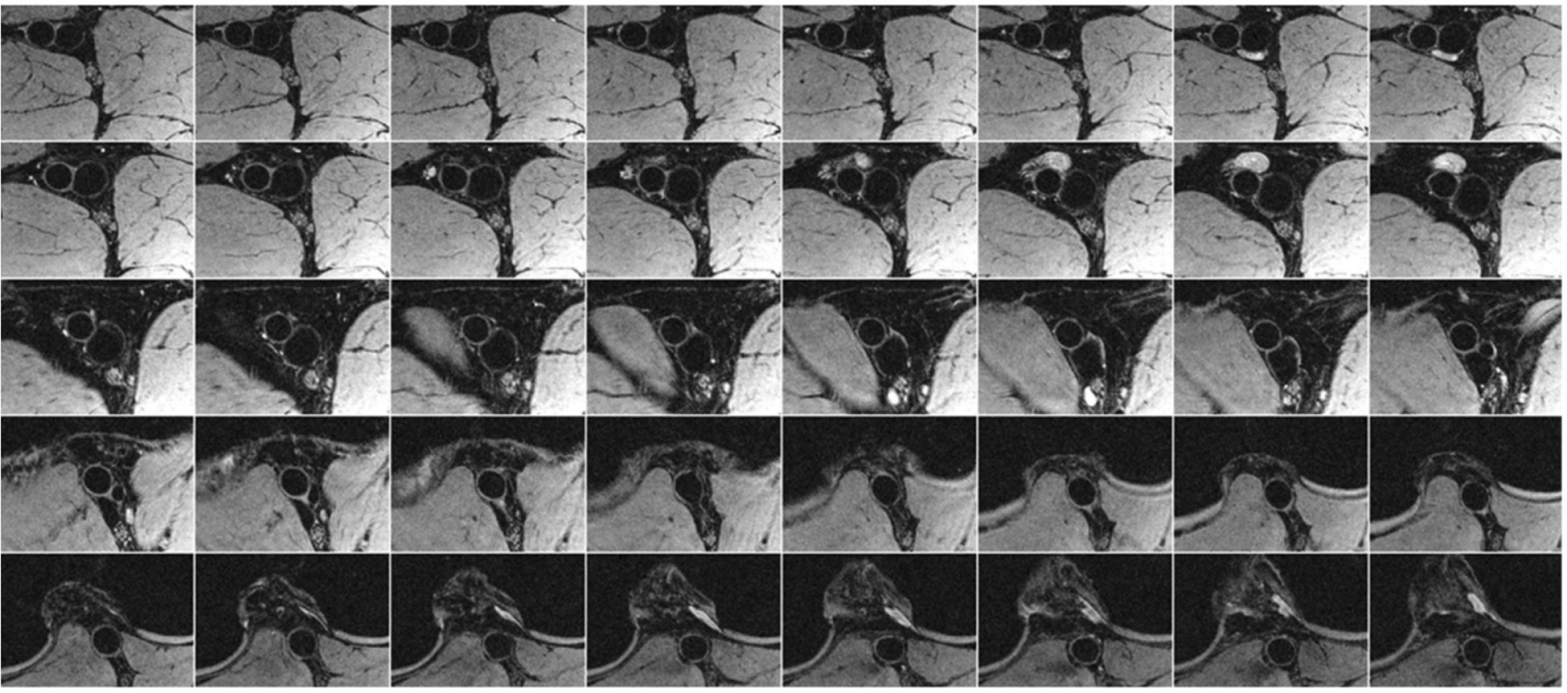
**Vessel-wall images of femoropopliteal artery and vein of a healthy 26 year-old subject.** Forty contiguous slices are shown in superior to inferior direction (left to right from the top left panel) direction. Larger transmural pressure of artery maintains circular cross section throughout the slices distinguishing artery from adjacent vein. We note that the blood signal suppression level is the same in artery and vein even though the velocity waveforms are quite different.

**Figure 5:**
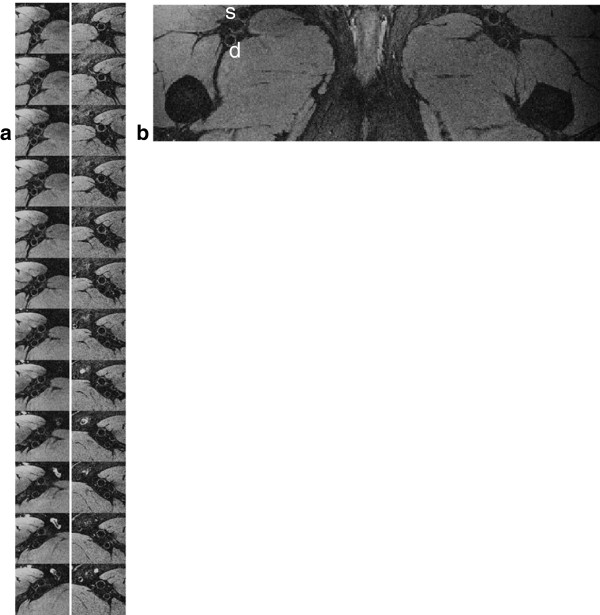
**Bilateral vessel-wall images of deep (d) and superficial (s) femoral arteries of a healthy 54 year-old subject. a)** Twelve contiguous slices (cropped) below the common femoral artery bifurcation; **b)** expanded view of fourth row in **a)**.

In general fat suppression was very effective with the 1–1 binomial pulse and allowed visualization of the vessel walls as shown by representative vessel-wall images of carotid arteries in Figure 3. In carotid imaging about 18 out of 25 slices were usable due to aliasing and limited coil sensitivity along the slab-direction. The typical SNR of the carotid vessel wall and lumen were 25 and 8, thus the average CNR between the wall and lumen was approximately 17. Average CNR efficiency over the entire vessel wall (usable slices only) was 8.2 mm^-3^ s^-1/2^. Figure 4 displays images of 40 contiguous slices (out of 96) of femoral and popliteal arteries acquired with the 8-channel extremity coil. Wall and lumen SNR were 19 and 5, respectively, resulting in CNR and CNR efficiency of 14 and 16.5 mm^-3^ s^-1/2^, respectively. In Figures 5a, b vessel-wall images of healthy deep and superficial femoral arteries acquired with body matrix and spine coils are shown. Typical vessel wall and lumen SNR were 6 and 2.5, respectively, giving CNR and CNR efficiency of 3.5 and 1.5 mm^-3^ s^-1/2^.

**Figure 6:**
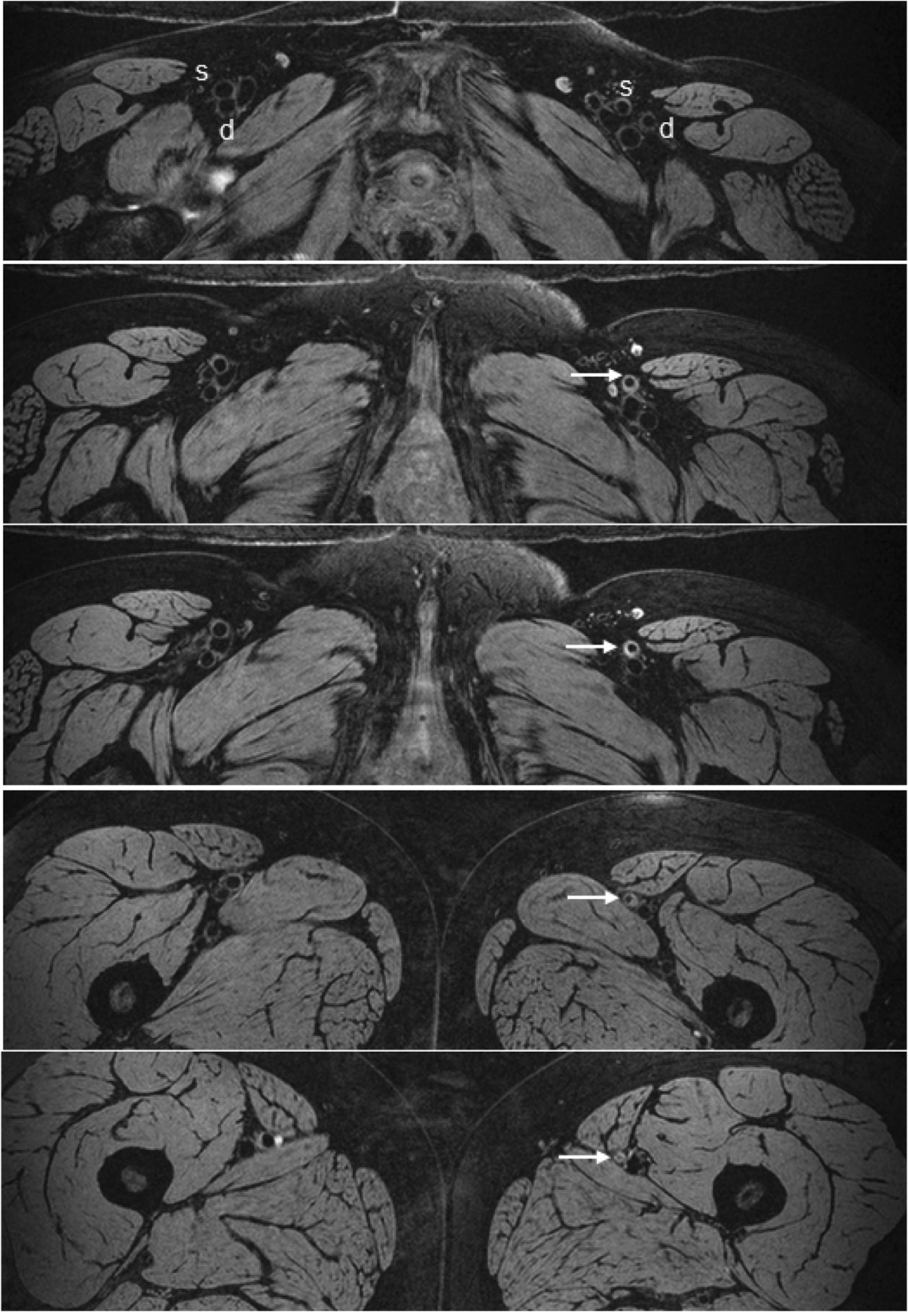
**Bilateral images of femoral arteries in a patient with unilateral PAD (ABI = 0.65 left leg, in comparison to disease-free right leg, ABI = 0.95).** Simultaneous acquisition of healthy and diseased arteries demonstrates the proposed method’s potential to visualize plaque.

In Figure 6 the differences in the plaque burden between the left (diseased) and right femoral arteries are highlighted. In the images of the first panel there is no evidence of atherosclerotic deposits in the arteries. At this level the SFA and DFA are visible bilaterally. In the next panel, the left SFA’s wall (white arrow) is noticeably thicker compared to the right femoral arteries. At more inferior locations (panels below) arrows point to progressive reduction of SFA lumen.

**Figure 7:**
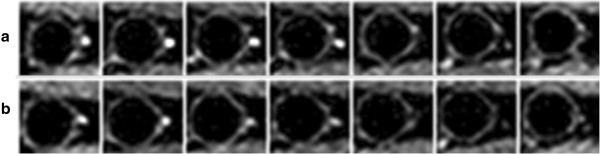
**FMD**_**A **_**of femoral artery.** All seven contiguous slices of a healthy 27 year-old right femoral artery at **a)** rest and **b)** 90s after the cuff deflation. In all slices the difference in lumen size representative of FMD_A_ is well visualized. The average estimated FMD_A_ is approximately 10%.

Figure 7 displays seven contiguous slices acquired at rest and during 90 s after the cuff deflation. These vessel-wall images (acquired in less than 12 s) demonstrate the potential application of the 3D SSFP-echo pulse sequence to assess endothelial function in peripheral arteries such as femoral or popliteal artery by quantifying FMD_A_. The average estimated FMD_A_ in seven slices was 10 ± 4%. The measured FMD_A_ of four subjects are summarized in Table 1. Qualitatively the FMD values and the age-related effects are consistent with those values estimated with ultrasound [28].

**Table 1:** Summary of the FMD measurements

**Age**	**FMD**_**A **_**(%)**
**27**	10
**33**	11
**41**	9
**54**	7

## Discussion

In this work we have described and demonstrated an alternative approach to 2D vessel-wall imaging of carotid and peripheral arteries using a 3D pulse sequence that collects flow-sensitive RF and stimulated echoes of SSFP, obviating the need for blood signal suppression preparation such as DIR, saturation or MSDE. Initial results indicate that the pulse sequence is equally sensitive to different types of flow waveform (monophasic and triphasic) and a wide range of blood flow velocities (including hyperemia in response to 5 mins of cuff-induced ischemia, Figure 7). The method will be useful for imaging the vessel walls of diseased peripheral arteries with more complex collateral circulation that may lead to a variety of flow patterns (mono-and bi-phasic velocity waveforms) and generally lower flow velocities. To achieve optimum blood signal suppression conventional methods (DIR and saturation) may need cardiac gating to ensure that “inverted” or “saturated” blood will have entered the imaging slice at the time of excitation and readout. Similarly, the MSDE technique is expected to be less effective during diastole due to limited flow-induced intravoxel dephasing because the average blood flow velocity across the lumen can be as low as 1 cm/s during the diastole in the femoral and popliteal circulation. However, the need for large FOV makes gating impractical given that coverage of the large vascular territory already requires a great deal more encoding steps than required for imaging the neck.

The work also highlights the method’s potential to quantify FMD_A_, a surrogate marker of endothelial function. In practice, the FMD_A_ of the artery can be monitored for the entire duration of hyperemia (~ 2-3 mins) in order to quantify maximum FMD_A_. Further, the proposed technique can be integrated with dynamic oximetry and velocimetry [29] to assess microvascular reactivity and FMD (surrogate marker of endothelial dysfunction) simultaneously with a single cuff paradigm. It should be noted that, to first order, FMD_A_ is twice that of FMD since ${\text{FMD}}_{A}=\frac{\mathrm{\delta A}}{A_{o}}=\frac{2\mathrm{\delta r}}{r_{o}}=2\frac{\mathrm{\delta r}}{r_{o}}=2\cdot \mathrm{FMD}$, where *δA*, *δr* and *r*_*o*_ represent incremental increase in the lumen area, incremental increase in the radius, initial area and initial radius, respectively. In short, FMD_A_ is more sensitive than a measurement of incremental diameter as in conventional FMD. Even though FMD can be quantified on the basis of bright-blood images the black-blood approach should have distinct advantages. The lumen area in bright-blood images is often underestimated, in particular in patients with atherosclerotic plaques. Perturbed flow downstream of stenoses and bifurcations can cause decreased phase coherence and thus loss of signal. Also, black-blood images are better suited for delineating the vessel wall against surrounding tissue for wall-area quantification.

A limitation of the pulse sequence underlying the present work is limited SNR resulting from the relatively long echo time (TE > TR),which was compensated by selecting a slice thickness more akin to that used in 2D imaging and, for carotid wall imaging, some signal averaging. On the other hand, the 8 CH extremity coil acquired images with higher in-plane resolution without averaging (Figure 4). The large coverage (~200 mm) of the extremity coil allows excitation of a wider slab (196 mm instead of 50 mm) taking advantage of the inherently greater SNR of 3D imaging (which scales with the square root of the number of slice encodings). Therefore, no signal averaging was needed for imaging superficial and deep femoral arteries. In contrast, the carotid coil was designed to acquire 2D images with limited coverage (~35 mm) along the slice direction. Another limitation is that SSFP-echo provides T2-weighting only whereas DIR with FSE readout can achieve T1, T2or PD weighting with appropriate TE and TR; different weighting allow characterization of plaque components [30]. As for any non-triggered vessel wall imaging technique, some reduction in SNR and possible blurring is expected. However, based on the observed sharpness of the images (for example Figure 4, femoropopliteal artery of a healthy young subject which is most pulsatile) the authors feel that possible further improvement in the quality of our 3D images does not warrant the trade-off of prolonged scan time exacted by cardiac gating.

Lastly, the effectiveness of the pulse sequence in comparison to current approaches requires further validation in patient studies.

## Conclusions

In conclusion, the proposed method is efficient, providing blood signal suppression largely independent of flow velocity, and is straightforward to implement. It is ideally suited for peripheral arteries where the average flow is low and large coverage is needed. As Zhang et al. [5] pointed out, vessel-wall imaging based on 2D multi-slice fast spin-echo would require over 30 mins for 360 mm peripheral-bed coverage that was used in our study. Further, SSFP-echo is a standard product pulse sequence that is commercially available. Another advantage of the method is the low SAR due to the small flip-angle. Lastly, the SSFP-echo pulse sequence can be implemented at 7T with minimal modification, e.g. the polarity of the slab-selective gradient have to be reversed for the 2nd RF to allow for shorter RF spacing to about 500 ms.

## Competing interests

The authors declare that they have no competing interests.

## Authors’ contributions

ML, EM, TF and FW conceived the study. ML analyzed and interpreted data. ML, CL, EE, EC and CL worked on the development of the CMR sequence and protocol. ML drafted the manuscript. All authors revised the manuscript critically for important intellectual content, read and approved the final manuscript.
